# Giant Descending Thoracic Aortic Aneurysm: A Case Report

**DOI:** 10.15388/Amed.2025.32.2.14

**Published:** 2025-12-30

**Authors:** Rassul Zhumagaliyev, Yerlan Orazymbetov, Serik Aitaliyev, Sidar Arslan, Tadas Lenkutis, Adakrius Siudikas, Rimantas Benetis

**Affiliations:** 1 Department of Cardiac, Thoracic and Vascular Surgery, Hospital of Lithuanian University of Health Sciences, Kauno Klinikos, Lithuanian University of Health Sciences, Kaunas, Lithuania; 2 Department of Cardiac, Thoracic and Vascular Surgery, Hospital of Lithuanian University of Health Sciences, Kauno Klinikos, Lithuanian University of Health Sciences, Kaunas, Lithuania; National Scientific Medical Center, Astana, Kazakhstan; 3 Department of Cardiac, Thoracic and Vascular Surgery, Hospital of Lithuanian University of Health Sciences, Kauno Klinikos, Lithuanian University of Health Sciences, Kaunas, Lithuania; Faculty of Medicine and Health Care, Al-Farabi Kazakh National University, Almaty, Kazakhstan; 4 Department of Cardiac, Thoracic and Vascular Surgery, Hospital of Lithuanian University of Health Sciences, Kauno Klinikos, Lithuanian University of Health Sciences, Kaunas, Lithuania; 5 Department of Cardiac, Thoracic and Vascular Surgery, Hospital of Lithuanian University of Health Sciences, Kauno Klinikos, Lithuanian University of Health Sciences, Kaunas, Lithuania; 6 Department of Cardiac, Thoracic and Vascular Surgery, Hospital of Lithuanian University of Health Sciences, Kauno Klinikos, Lithuanian University of Health Sciences, Kaunas, Lithuania; 7 Department of Cardiac, Thoracic and Vascular Surgery, Hospital of Lithuanian University of Health Sciences, Kauno Klinikos, Lithuanian University of Health Sciences, Kaunas, Lithuania

**Keywords:** giant descending thoracic aortic aneurysm (GDTAA), aortic dilation, surgical repair, didelė nusileidžiančiosios krūtinės aortos aneurizma (NKAA), aortos išsiplėtimas, chirurginis gydymas

## Abstract

**Background:**

Giant descending thoracic aortic aneurysm (GDTAA) is a rare vascular disease characterized by an aortic diameter exceeding 10 cm. GDTAA carries a significant risk of rupture and mortality and requires timely diagnosis and intervention. Despite the clinical severity of the disease, the literature on GDTAA remains sparse, particularly in cases with extreme aneurysmal dilatation.

**Case Presentation:**

We present the case of a 68-year-old man with a GDTAA of 14.08 × 10.04 cm, one of the largest ever reported. The patient initially presented with recurrent syncope, chronic cough and fatigue. Imaging studies, including *Computed Tomography* (CT) angiography, revealed a massive aneurysmal dilatation in the distal post-arch segment of the descending aorta with compression of the trachea and bronchi. The patient underwent a successful open surgical repair with a Dacron graft and simultaneous *Coronary Artery Bypass Grafting* (CABG). Postoperative complications included respiratory acidosis, emphysema and transient haemodynamic instability, which were effectively treated. The patient was discharged in a stable condition on the tenth postoperative day.

**Conclusion:**

This case highlights the importance of early recognition and surgical intervention in GDTAA in order to prevent catastrophic consequences. Comprehensive preoperative evaluation, careful surgical planning and attentive postoperative care are essential for optimal recovery. Our results emphasise the importance of modern imaging techniques for accurate anatomical assessment and risk stratification in patients with extreme aneurysm growth. Further research is needed to establish standardised protocols for the treatment of GDTAA.

## Abstract (it)

**Įvadas:**

Didelė nusileidžiančiosios krūtinės aortos aneurizma (NKAA) yra reta kraujagyslių patologija, kai aortos skersmuo viršija 10 cm. Ši aneurizma pasižymi didele plyšimo ir mirtingumo rizika, todėl laiku atlikta diagnostika ir chirurginis gydymas yra gyvybiškai svarbūs. Literatūroje didelė NKAA aprašoma retai, ypač tokie atvejai, kai aneurizma siekia 10 cm ir daugiau.

**Atvejo pristatymas:**

68 metų vyrui diagnozuota didelė NKAA, ji buvo 14,08×10,04 cm – tai vienas iš retų literatūroje aprašytų atvejų. Pacientas kreipėsi į gydymo įstaigą dėl pasikartojančių sinkopių, lėtinio kosulio ir nuovargio. Vaizdiniai tyrimai, įskaitant kompiuterinę tomografiją (KT) su angiografija, parodė esant didžiulę aneurizmą distaliojoje aortos lanko dalyje, sukėlusią trachėjos ir bronchų spaudimą. Pacientui sėkmingai atlikta atvira chirurginė rekonstrukcija, naudojant *Dacron* protezą, kartu atliktas ir vainikinių arterijų šuntavimas (AKŠ). Pooperacines komplikacijas – respiracinę acidozę, emfizemą ir laikiną hemodinaminį nestabilumą pavyko sėkmingai suvaldyti. Pacientas dešimtą pooperacinę parą išrašytas stabilios būklės.

**Išvados:**

Šis atvejis pabrėžia ankstyvosios didelės NKAA diagnostikos ir chirurginio gydymo svarbą, siekiant išvengti sunkių padarinių. Išsamus priešoperacinis įvertinimas, kruopštus chirurginis planavimas ir atidi pooperacinė priežiūra yra būtini siekiant optimalių rezultatų. Ypač svarbi pažangių vaizdinių tyrimų metodų reikšmė tiksliai pacientų, kuriems nustatyta didelė aortos aneurizma, anatomijos analizei ir rizikos stratifikacijai. Reikia tolesnių tyrimų, siekiant nustatyti standartizuotus didelių NKAA gydymo protokolus.

## Introduction

Giant descending thoracic aortic aneurysm (GDTAA) is an extremely rare vascular disease with limited representation in the existing literature due to its rarity [1]. Although various studies have provided detailed definitions and classifications, GDTAA is typically characterised when the maximum transverse diameter of the aorta exceeds 10 cm, regardless of sex. While most patients with symptomatic aneurysms are diagnosed before they reach such significant proportions, asymptomatic individuals can reach extreme proportions without complications [2]. Although the reported incidence of GDTAA varies geographically, the overall average prevalence of thoracic aortic aneurysms is approximately 10 per 100,000 individuals [3].

The diagnosis of GDTAA requires a thorough differential diagnostic approach to differentiate it from conditions such as *Acute Coronary Syndrome* (ACS), valvular heart disease or typical heart failure symptoms, all of which may have overlapping clinical features that are influenced by the size of the aneurysm [4]. Due to the chronic nature and variable presentation of the disease, the characteristics of pain during acute episodes are often considered unreliable for distinguishing between ACS and thoracic aortic aneurysm [5, 6]. In this context, a comprehensive evaluation of the patient’s medical history plays a crucial role in the selection of appropriate diagnostic tools, such as *Computed Tomography* (CT), especially in emergency situations where timely diagnosis is crucial to reduce mortality. For example, a history of myocardial infarction and diabetes are strongly associated with ACS.

Patients with descending thoracic aortic aneurysms less than 6 cm in diameter may be considered for surgical intervention if they have an underlying genetic disorder (Marfan, Ehlers-Danlos, Loeys-Dietz or Turner syndrome) [7]. Without this genetic predisposition, usually recommended conservative treatment. Elective surgical repair is generally indicated if the aneurysm has a transverse diameter of more than 6.5 cm. If the aneurysm is expanding at a rate of more than 1 cm per year, earlier surgical intervention may be warranted, even for smaller diameters, so that to reduce the risk of rupture or dissection [8].

The clinical presentation of giant thoracic aortic aneurysms is primarily due to the mass effect of the aneurysm and less to heart failure or aortic regurgitation. As the aneurysm enlarges, it can compress the surrounding anatomical structures, including the left recurrent laryngeal, phrenic, and vagus nerves, resulting in persistent symptoms such as nausea and cough that are unresponsive to medication. In addition, significant airway compression can mimic features of obstructive lung disease, manifesting as dyspnoea, wheezing, and chest discomfort [9].

A rare but life-threatening complication of GDTAA is the formation of aortoesophageal or aortotracheal fistulas. These fistulas are observed more frequently in large aneurysms than in smaller aneurysms. Their formation may be related to underlying pathological conditions such as oesophageal carcinoma, mediastinal tuberculosis, trauma or postoperative complications that lead to abnormal connections between the aorta and adjacent structures such as the oesophagus. Without surgical intervention, the prognosis is consistently fatal, with a reported mortality rate of 100%, while surgical repair is associated with a high perioperative mortality of 30 to 80% [10]. In addition, in patients with chronic leukaemia, leukaemic infiltration of the mediastinal tissue surrounding a giant aneurysm can complicate the condition, potentially increasing the risk of severe vascular complications if not addressed promptly [11].

The present clinical case describes a patient with GDTAA, distinguished by one of the largest recorded transverse diameters at the time of diagnosis, accompanied by an elevated initial risk of rupture and mortality.

## Case Presentation

### Medical History

A 68-year-old male patient with a medical history of stage 2 hypertension and dyslipidemia presented to the cardiology clinic due to recurrent episodes of syncope. Upon admission, the patient also reported symptoms of cough and fatigue. The patient’s medical history was notable for chronic B-cell lymphocytic leukemia, which was in remission at the time of admission to the cardiac surgery department. Surgical history included previous operations for appendicitis and gastric perforation. The patient had a 45-year history of smoking, limited to 2–3 cigarettes per day. Social history was unremarkable, with no family history of aortic aneurysms or genetic disorders to be noted. Family history included hypertension in first-degree relatives but no documented vascular diseases.

### Clinical Findings

Physical examination findings were unremarkable, with no audible bruits, masses, or neurological deficits. Vital signs on admission were as follows: blood pressure 150/90 mmHg, heart rate 60 bpm, respiratory rate 18/min, oxygen saturation 96% on room air.

### Diagnostic Evaluations

**Figure 1:**
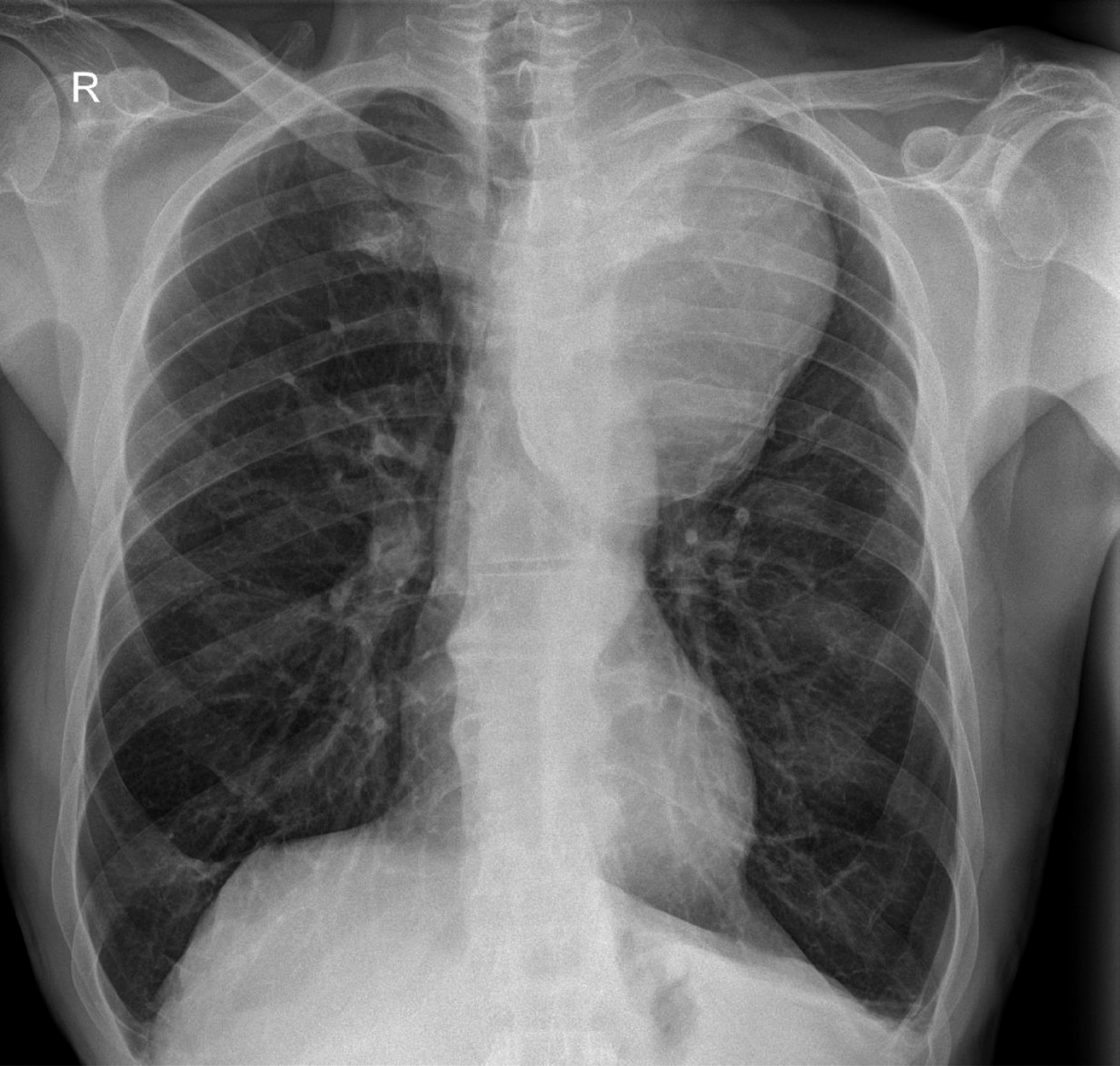
Pre-op chest X-ray with massive mediastinal mass with trachea deviated to the right

- Given the clinical presentation suggestive of heart failure or syncope, coronary angiography and *Transthoracic Echocardiography* (TTE) were performed.
- Coronary angiography revealed significant stenosis in two coronary arteries: the right main coronary artery and the mid-*Left Anterior Descending* (LAD) artery.
- Echocardiographic findings: The aortic valve was tricuspid with moderate sclerosis. Mild (grade 1) regurgitation was observed in both the mitral and tricuspid valves. Left ventricular function was preserved, with an ejection fraction (EF) of 55%. Left ventricular mass index (LVMI) was measured at 107.34 g/m^2^, whereas the *Left Ventricular Mass* (LVM) was calculated as 207.17 g, indicating a tendency toward hypertrophy. The right heart chambers were within normal limits without evidence of dilation, and there were no signs of pulmonary hypertension. The calculated EuroSCORE II was 2.49.
- Preoperative and postoperative *Electrocardiograms* (ECG) did not reveal any significant changes that could affect clinical outcomes. However, the ECG prior to discharge indicated sinus bradycardia, ST-segment abnormalities in the inferior and lateral segments, and notable left ventricular hypertrophic changes.
- Posteroanterior and lateral chest radiographs demonstrated no pathological findings in the pleural spaces or lung parenchyma. However, mediastinal imaging identified fibrotic lesions with deformation, likely secondary to previous radiotherapy. The most striking observation was a large mass in the region of the descending aorta, situated between the pulmonary shadows, with rightward displacement of the trachea. Figure 1 illustrates the preoperative chest X-ray, highlighting the massive formation in the left mediastinal region.

**Figure 2:**
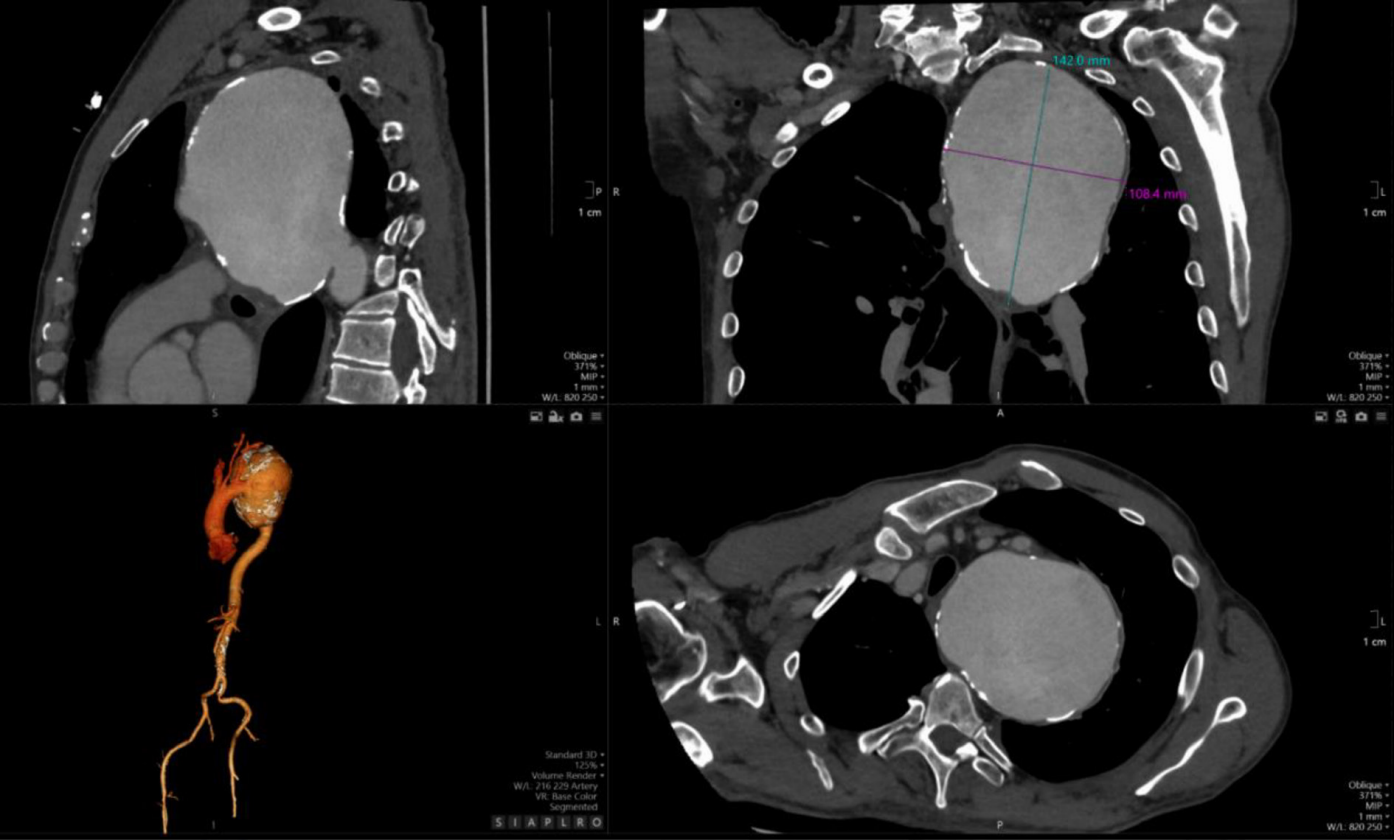
2-D CT, showing sagittal, oblique transverse section of aneurysmal thoracic aorta

The detection of a mediastinal mass prompted differential diagnostic considerations, primarily focusing on two major possibilities: (1) mediastinal tumor formation associated with lymphocytic infiltration due to leukemia or other oncological masses, and (2) a giant vascular aneurysm. To delineate the etiology, a two-dimensional CT scan of the thoracic aorta was performed, as shown in Figure 2, revealing a large aneurysm in the distal post-arch segment of the descending aorta. The observed mass corresponds to a giant aneurysmal dilation.

**Figure 3:**
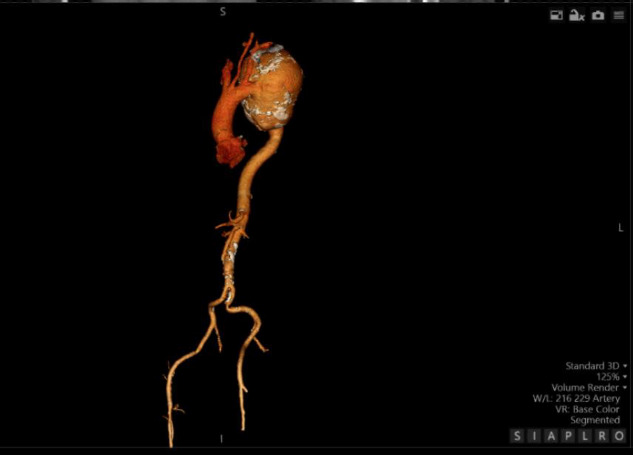
Detailed 3-D CT scan of aortic arch and the rest of the aorta

The aneurysm measured 14.08 × 10.04 cm in its maximum transverse diameters. No chronic thrombus was identified within the tunica media, nor was there any evidence of dissection. The proximal ascending aorta measured 38 mm in diameter. Figure 3, which depicts a three-dimensional CT reconstruction, delineates the anatomical relationship of the aneurysm with the aortic arch, the left subclavian artery, and the descending thoracic aorta. The aneurysm was localized, with no involvement of the proximal left subclavian artery or its orifice. The distal portion of the aneurysm did not extend to spinal or intercostal branches. The remainder of the aorta, including the abdominal segment, appeared normal. The primary clinical impact of the aneurysm was attributed to a significant mass effect, leading to compression of the trachea and bronchi as well as potential irritation of the left phrenic nerve secondary to mediastinal compression.

## Treatment

The patient was prepared for surgery under standard cardiac anesthesia and positioned in the prone position. A median sternotomy was performed, followed by pericardiectomy. Cannulation of the aorta and right atrium was achieved, and cold crystalloid cardioplegia was administered antegradely via the ascending aorta. Dissection of the aortic arch and descending aorta was undertaken, spanning from the left subclavian artery to the descending thoracic aorta. Critical anatomical structures, including the left phrenic nerve, extensions of the left recurrent laryngeal nerve, and the vagus nerve, were carefully identified and preserved through blunt dissection.

Aneurysmectomy was executed with aortic incision (aortotomy), followed by intraluminal inspection, which confirmed the absence of involvement of spinal arteries or the thoracic segment. Macroscopically, the aneurysm wall appeared thinned and dilated without visible intramural thrombus or dissection flaps. No histological examination of the resected aortic specimen was performed, as it was not deemed necessary for clinical management; the etiology was presumed to be atherosclerotic based on the patient’s risk factors (hypertension, dyslipidemia, smoking), though idiopathic or degenerative causes could not be ruled out. A proximal anastomosis was performed by using a 30 mm Dacron graft with 4-0 Prolene sutures at the border of the left subclavian artery. The distal anastomosis was executed in a continuous fashion with 4-0 Prolene sutures to the descending aorta at the terminus of the aneurysmal segment. Following the completion of aortic grafting, *Coronary Artery Bypass Grafting* (CABG) was performed, targeting segments S7–S9.

## Postoperative Course

The patient was transferred to the *Intensive Care Unit* (ICU) for postoperative monitoring. The early postoperative course was complicated by respiratory acidosis, observed on the second postoperative day along with emphysema and pulmonary edema. Hemodynamic instability due to significant blood loss was noted on postoperative day 2, with a total blood loss of 800 mL. Despite the volume loss, resternotomy was not pursued. The patient experienced profound hypovolemia, necessitating vasopressor support with norepinephrine administered as a continuous infusion at a rate of 0.04 μg/kg/min. Following the stabilization of the acid-base balance, the patient was extubated on the second postoperative day.

On the fourth postoperative day, the patient was transferred back to the cardiac surgery department in stable condition. On the tenth postoperative day, the patient was discharged for outpatient follow-up after the completion of the rehabilitation program.

## Discussion

GDTAA, defined as aneurysms exceeding 10 cm in diameter, represents an exceptionally rare and clinically challenging condition, with an estimated prevalence of 10 per 100,000 individuals in the general population [3]. Despite significant advances in diagnostic and therapeutic modalities, GDTAA continues to carry a high risk of morbidity and mortality due to its size and anatomical involvement [1]. The presented case of a 68-year-old male with a 14.08 × 10.04 cm GDTAA exemplifies the critical need for an early detection and timely intervention so that to prevent catastrophic complications such as rupture and dissection [7].

The pathogenesis of GDTAA is associated with degenerative changes in the media, leading to progressive weakening and dilation of the aortic wall under chronic hemodynamic stress [12]. Histological analyses typically indicate that fragmentation of elastic fibers and smooth muscle cell loss contribute to the pathological expansion of the vessel; common features include cystic medial degeneration, atherosclerosis, or inflammation, though, in this particular case, no histology was performed to confirm the assumption [13]. Key risk factors include long-standing hypertension, dyslipidemia, smoking, and genetic predispositions such as Marfan syndrome, Ehlers-Danlos syndrome, and Loeys-Dietz syndrome [14]. In our case, the history of hypertension and dyslipidaemia associated with aneurysmal growth was exacerbated by chronic B-cell lymphocytic leukaemia, which has been reported to complicate aortic aneurysms through potential infiltration or associated vascular degeneration [15, 16].

GDTAA expansion is influenced by haemodynamic forces that exacerbate elastic fibre degeneration and compromise the vascular smooth muscle integrity [12]. An increased wall stress caused by systemic hypertension and local pressure gradients accelerates pathological remodelling of the aortic wall [17]. Recent studies also suggest that chronic inflammation through macrophage infiltration and cytokine release plays a crucial role in the progression of thoracic aortic aneurysms [18].

GDTAA often presents with non-specific symptoms that are primarily due to mass effect rather than rupture [19]. Common clinical manifestations include dyspnoea, cough, chest pain and dysphagia due to compression of adjacent structures such as the trachea, bronchi and oesophagus [9]. In the case presented, the patient had recurrent syncope, persistent cough and fatigue, which correlated with significant deviation of the trachea and compression of the airway structures on imaging [20].

CT angiography remains the gold standard for the diagnosis and assessment of the extent of GDTAA [21]. It allows high-resolution visualisation of the size and morphology of the aneurysm as well as the anatomical relationships with the adjacent structures [21]. In this case, CT imaging confirmed a massive aneurysm with significant displacement of the trachea and compression of the bronchi, thereby emphasising the crucial role of modern imaging in surgical planning [21].

Surgical intervention is the primary method of treating GDTAA, especially if the aneurysm is larger than 6.5 cm or symptomatic [22]. Open surgical repair, as performed in this case, involves resection of the aneurysmal segment and replacement with a Dacron graft, which has been shown to be durable over time [23]. *Thoracic Endovascular Aortic Repair* (TEVAR) is a new alternative that is associated with less perioperative morbidity and faster recovery [3]. However, its applicability is limited in cases of an extreme aneurysm size or unfavourable anatomical configurations [24].

In the presented case, open surgical repair was chosen due to the giant size of the aneurysm and its anatomical complexity, which would have posed significant challenges for endovascular stent placement [24]. The simultaneous performance of *Coronary Artery Bypass Grafting* (CABG) is another example of the need to consider concomitant cardiovascular conditions when repairing an aortic aneurysm [25].

To provide the context, the following table compares the key clinical characteristics of selected published GDTAA case reports with the present case:

**Table 1:** Summary of Selected Giant Descending Thoracic Aortic Aneurysm Cases (>10 cm)

Reference	Age/Sex	Location	Size (cm)	Symptoms	Treatment
Present case	68/M	Descending thoracic	14.08 × 10.04	Recurrent syncope, chronic cough, fatigue	Open repair with Dacron graft + CABG
González-Urquijo et al. (2018) (1)	Not specified	Thoracic	15	Asymptomatic	Endovascular repair
Okura et al. (1999) (2)	Not specified	Thoracic	Giant (not quantified)	Not specified (unruptured)	Not specified
Oh et al. (2020) (5)	48/M	Descending thoracic	12	Squeezing chest pain mimicking ACS	TEVAR
De Mora et al. (2022) (4)	42/M	Ascending thoracic and arch	Giant (not quantified)	Not specified	Not specified

This comparison highlights the variability in presentation and management; our case stands out for its extreme size and the combined open surgical approach in a symptomatic patient with comorbidities.

Postoperative complications following GDTAA repair may include respiratory failure, bleeding, haemodynamic instability and, in rare cases, paraplegia due to spinal cord ischaemia [26, 27]. The patient experienced transient respiratory acidosis, emphysema and pulmonary oedema after the surgery, which were effectively treated and led to a favourable outcome [28]. Despite these challenges, the patient was discharged on the tenth postoperative day, thus emphasising the importance of careful perioperative management [29].

## Conclusion

The limited literature on GDTAA highlights the need for centre-specific treatment protocols developed in collaboration with interventional cardiology and adapted to current guidelines. A better understanding of the pathophysiology of GDTAA, including the role of genetic and haematological conditions (such as chronic B-cell lymphocytic leukemia in this case, which may contribute to vascular weakening or complications), is essential for early risk stratification and timely intervention. New imaging techniques such as 3D CT provide valuable insights into anatomical relationships and support more precise diagnosis and treatment planning.

## Data Availability

All data generated during this study are included in this article. Further inquiries can be directed to the corresponding author.

## Abbreviations

GDTAA – Giant Descending Thoracic Aortic Aneurysm

CABG – Coronary Artery Bypass Grafting

CT – Computed Tomography

TTE – Transthoracic Echocardiography

ICU – Intensive Care Unit

ECG – Electrocardiogram

EF – Ejection Fraction

LVMI – Left Ventricular Mass Index

LVM – Left Ventricular Mass

TEVAR – Thoracic Endovascular Aortic Repair

SVC – Superior Vena Cava

CSF – Cerebrospinal Fluid

3D CT – Three-Dimensional Computed Tomography

LV – Left Ventricle

RV – Right Ventricle

## Santrumpos

GDTAA – Milžiniška nusileidžiančios krūtinės aortos aneurizma

CABG – Arterijos koronarinės šuntavimas

CT – Kompiuterinė tomografija

TTE – Transtorakalinė echokardiografija

ICU – Intensyviosios terapijos skyrius

ECG – Elektrokardiograma

EF – Išmetimo frakcija

LVMI – Kairiojo skilvelio masės indeksas

LVM – Kairiojo skilvelio masė

TEVAR – Torakalinė endovaskulinė aortos rekonstrukcija

SVC – Viršutinė tuščioji vena

CSF – Cerebrospinalinis skystis

3D CT – Trimatė kompiuterinė tomografija

LV – Kairysis skilvelis

RV – Dešinysis skilvelis

## References

[1] González-Urquijo M, Dominguez-Porras VA, Tellez-Martinez LG, Lozano-Balderas G, Flores-Villalba E, Fabiani MA (2018). A case report of successful endovascular repair of a giant 15 cm diameter asymptomatic thoracic aortic aneurysm. *Int J Surg Case Rep*.

[2] Okura T, Kitami Y, Takata Y, Fukuoka T, Arimitsu J, Hiwada K (1999). Giant unruptured aneurysm of the thoracic aorta. A case report. *Angiology*.

[3] Makaroun MS, Dillavou ED, Wheatley GH, Cameron DE (2005). Endovascular treatment of thoracic aortic aneurysms: Results of the phase II multicenter trial of the GORE TAG thoracic endoprosthesis. *J Vasc Surg*.

[4] De Mora V, Yic C, Servente LT (2022). Aneurisma gigante de aorta torácica. A propósito de un caso clínico. *Rev Colomb Radiol*.

[5] Oh S, Cho KH, Kim MC, Jeong MH, Kim JH (2020). A case of a gigantic thoracic aortic aneurysm initially mimicking acute coronary syndrome and treated endovascularly. *Korean J Intern Med*.

[6] Alhatemi AQM, Aziz EMH, Al-Yaseen AHA (2024). Aortic Aneurysm Mimicking Inferior ST-Elevation Myocardial Infarction: A Case Report. *J Investig Med High Impact Case Rep*.

[7] Hiratzka LF, Bakris GL, Beckman JA (2010). ACCF/AHA/AATS/ACR/ASA/SCA/SCAI/SIR/STS/SVM guidelines for the diagnosis and management of patients with thoracic aortic disease: Executive summary. *Circulation*.

[8] Findeiss LK, Cody ME (2011). Endovascular Repair of Thoracic Aortic Aneurysms. *Semin Intervent Radiol*.

[9] Mitsali T, Dewi DK, Hilman (2025). Giant ascending aortic aneurysm: A rare case report. *Radiol Case Rep*.

[10] da Silva ES, Tozzi FL, Otochi JP, de Tolosa EM, Neves CR, Fortes F (1999). Aortoesophageal fistula caused by aneurysm of the thoracic aorta: successful surgical treatment, case report, and literature review. *J Vasc Surg*.

[11] Yang Y, Hu D, Peng D (2018). Primary aortoesophageal fistula: a fatal outcome. *Am J Emerg Med*.

[12] Faiza Z, Sharman T (2023). Thoracic Aorta Aneurysm. StatPearls [Internet].

[13] Isselbacher EM (2005). Thoracic and Abdominal Aortic Aneurysms. *Circulation*.

[14] Isselbacher EM, Caughey MC, Starnes BW (2019). Optimal medical therapy for thoracic aortic aneurysms: A comparative review. *Am J Cardiol*.

[15] Schiffrin EL, Touyz RM (2018). Vascular Biology of Hypertension. Hypertension.

[16] Larson RA, Anastasi J (2011). Acute lymphoblastic leukemia: diagnosis and classification. *Best Pract Res Clin Haematol*.

[17] Humphrey JD, Schwartz MA, Tellides G, Milewicz DM (2015). Role of Mechanotransduction in Vascular Biology: Focus on Thoracic Aortic Aneurysms and Dissections. *Circ Res*.

[18] Dale MA, Ruhlman MK, Baxter BT (2015). Inflammatory cell phenotypes in AAAs: their role and potential as targets for therapy. *Arterioscler Thromb Vasc Biol*.

[19] Coady MA, Rizzo JA, Hammond GL (1997). What is the appropriate size criterion for resection of thoracic aortic aneurysms?. *J Thorac Cardiovasc Surg*.

[20] Leitman IM, Suzuki K, Wengrofsky AJ (2013). Early recognition of acute thoracic aortic dissection and aneurysm. *World J Emerg Surg*.

[21] Bavare CS, Amaral JF (2020). Risk Stratification in Thoracic Aortic Aneurysm Surgery. *J Thorac Cardiovasc Surg*.

[22] Elefteriades JA, Sang AX, Kuzmik GA (2015). Indications and timing of repair for thoracic aortic aneurysms. *J Thorac Cardiovasc Surg*.

[23] Svensson LG, Kouchoukos NT, Miller DC (2008). Expert consensus document on the treatment of descending thoracic aortic disease using endovascular stent-grafts. *Ann Thorac Surg*.

[24] Hughes GC, Shah AA, Williams JB (2013). Thoracic endovascular aortic repair (TEVAR) for chronic aortic dissection: Results of a prospective intent-to-treat study. *J Thorac Cardiovasc Surg*.

[25] Patel HJ, Williams DM, Upchurch GR (2021). Thoracic endovascular aortic repair: Lessons learned and implications for the future. *J Vasc Surg*.

[26] Wanhainen A, Verzini F, Van Herzeele I (2022). Editor‘s Choice - European Society for Vascular Surgery (ESVS) 2022 Clinical Practice Guidelines on the Management of Aortic Aneurysms. *Eur J Vasc Endovasc Surg*.

[27] Coselli JS, LeMaire SA, Conklin LD, Adams GJ (2004). Left heart bypass during descending thoracic aortic aneurysm repair: a clinical study. *Ann Thorac Surg*.

[28] Hughes GC (2013). Management of thoracoabdominal aortic aneurysms. *Cardiovasc Surg*.

[29] Safi HJ, Miller CC (1999). Spinal cord protection in descending thoracic and thoracoabdominal aortic repair. *Ann Thorac Surg*.

